# Targeting the VEGF-C/VEGFR3 axis suppresses Slug-mediated cancer metastasis and stemness via inhibition of KRAS/YAP1 signaling

**DOI:** 10.18632/oncotarget.13629

**Published:** 2016-11-25

**Authors:** Yu-Wen Yeh, Ching-Chia Cheng, Shu-Ting Yang, Chi-Feng Tseng, Ting-Yu Chang, Sin-Ying Tsai, Earl Fu, Chien-Ping Chiang, Li-Chuan Liao, Pei-Wen Tsai, Yung-Luen Yu, Jen-Liang Su

**Affiliations:** ^1^ Division of Dermatology, Tri-Service General Hospital Songshan Branch, National Defense Medical Center, Taipei 10581, Taiwan; ^2^ Graduate Institute of Medical Sciences, National Defense Medical Center, Taipei 11490, Taiwan; ^3^ Graduate Institute of Life Sciences, National Defense Medical Center, Taipei 11490, Taiwan; ^4^ National Institute of Cancer Research, National Health Research Institutes, Zhunan, Miaoli County 35053, Taiwan; ^5^ Department of Biotechnology, Asia University, Taichung 41354, Taiwan; ^6^ Department of Periodontology, Tri-Service General Hospital, National Defense Medical Center, Taipei 11490, Taiwan; ^7^ Department of Dermatology, Tri-Service General Hospital, National Defense Medical Center, Taipei 11490, Taiwan; ^8^ Bioresource Collection and Research Center, Food Industry Research and Development Institute, Hsinchu 30062, Taiwan; ^9^ Institute of Molecular and Cellular Biology, National Tsing Hua University, Hsinchu 30013, Taiwan; ^10^ Graduate Institute of Biomedical Sciences, China Medical University, Taichung 40402, Taiwan; ^11^ Center for Molecular Medicine, China Medical University Hospital, Taichung 40447, Taiwan

**Keywords:** VEGF-C, YAP1, metastasis, cancer stemness, skin cancer

## Abstract

Vascular endothelial growth factor-C (VEGF-C) has been implicated in epithelial-mesenchymal transition (EMT) processes and various human cancers, including skin cancer. Skin cancer is an aggressive human malignancy with increasing incidence worldwide; however, the underlying mechanisms involved in VEGF-C-induced skin cancer stemness and metastasis remain unclear. Here, we report that VEGF-C enhances skin cancer migration, invasion and stemness through Slug up-regulation. Oncomine database analysis indicated that the KRAS/MAPK (mitogen-activated protein kinases) pathway and YAP1 (yes-associated protein 1) expression are positively correlated with metastatic skin cancer. We show that VEGF-C triggers the activation of KRAS/MAPK signaling to increase YAP1 and downstream Slug expression, which are suppressed by an anti-VEGFR3 (VEGF receptor 3) peptide, a specific peptide targeting VEGFR3. The VEGF-C-induced migration, invasion and stemness of skin cancer cells are also abrogated by the anti-VEGFR3 peptide. Based on these data, we reveal the role of the VEGF-C/VEGFR3-mediated KRAS/MAPK-YAP1/Slug pathway in skin cancer progression and propose that the VEGF-C/VEGFR3 axis is a promising target for the anti-VEGFR3 peptide.

## INTRODUCTION

Skin cancers are classified into three types, melanoma, squamous cell carcinoma (SCC) and basal cell carcinoma (BCC), with SCC and BCC known as non-melanoma skin cancer (NMSC) [1, 2]. BCC accounts for approximately 80% of all non-melanoma skin cancers and is the most common skin cancer [3]. Despite the increasing incidence of BCC, metastasis of this type of cancer is rare, occurring in 0.0028% to 0.55% of all BCC cases [4, 5]. Since metastatic BCC (MBCC) is uncommon, the lack of available information regarding this type of cancer is expected. MBCC usually spreads by lymphatic and hematogenous routes [6], and the 5-year survival of patients with metastatic or advanced BCC is only 10% [7], whereas the 10-year survival rate for patients with metastatic melanoma is less than 10% [8]. Therefore, investigating the underlying mechanisms involved in metastasis, invasion and progression of skin cancers is important for the development of novel therapeutic strategies. The vascular endothelial growth factor (VEGF) family consists of VEGF-A, -B, -C, -D, -E, -F and placental growth factor (PlGF), which have been identified as specific angiogenic and lymphangiogenic factors in tumor progression and metastasis [9, 10]. The cellular functions of the VEGFs are mediated by three tyrosine kinase receptors (VEGFRs), VEGFR1, 2 and 3 [11]. Among these VEGF-VEGFR complexes, VEGF-C binds to both VEGFR2 and VEGFR3 and further enhances diverse biological effects, such as cell growth, proliferation, mobility and invasiveness, to promote angiogenesis, metastasis and tumor progression [12–14]. Previous studies reported that activation of the VEGF-C/VEGFR3 signaling pathway increases cell migration and invasion to promote cancer metastasis, and a high expression level of VEGF-C has been correlated with the shortest survival time in lung cancer [10, 15] and esophageal squamous cell carcinoma [16, 17]. Elevated VEGF-C expression is also found in the lymph nodes of patients with metastatic melanoma [18]. In addition, VEGF-C has been recognized as a metastatic tumor promoter by protecting tumor cells against preexisting antitumor immunity and contributes to tumor progression of melanoma cells [19]. However, how VEGF-C is involved in cell mobility and the progression of skin cancer has remained elusive.

Yes-associated protein 1 (YAP1), a downstream molecule of the Hippo signaling pathway, has been identified as an oncogene involved in cancer-promoting processes such as cell proliferation and metastasis [20–22]. YAP1 interacts with various transcription factors, such as RUNX2, p73, p53BP2 and TEAD family members, that are essential for YAP1-mediated tumor growth and metastasis in melanoma and breast cancer [21, 23]. In addition, it has been reported that elevated YAP1 regulates epithelial-mesenchymal transition (EMT) of the atrioventricular cushion [24] and expands multipotent undifferentiated progenitor cells [25], suggesting that YAP1 plays a critical role in promoting cell metastasis, organ growth, cancer stemness and morphological changes. For now, it is still unclear whether YAP1 is involved in the VEGF-C/VEGFR3 signaling pathway in skin cancers.

In this study, we demonstrate that VEGF-C/VEGFR3 signaling affects skin cancer cell mobility by mediating Slug expression. VEGF-C regulates CSC properties and is positively correlated with metastasis and the expression of CSC markers in skin cancer. We further identify VEGF-C/VEGFR3-mediated YAP1 and Slug expression through KRAS/MAPK signaling in skin cancer. By treating skin cancer cells with an anti-VEGFR3 specific peptide [26], we show that suppression of VEGF-C/VEGFR3 signaling significantly reduces YAP1 and Slug expression and diminishes the migration, invasion and cancer stemness of the cells. In conclusion, our findings provide a novel signaling pathway, the VEGF-C/VEGFR3-KRAS/MAPK-YAP1/Slug axis, in skin cancer progression and a potential therapeutic strategy by treatment with an anti-VEGFR3 peptide.

## RESULTS

### VEGF-C increases the migration and invasion abilities of skin cancer cells

**Figure 1:**
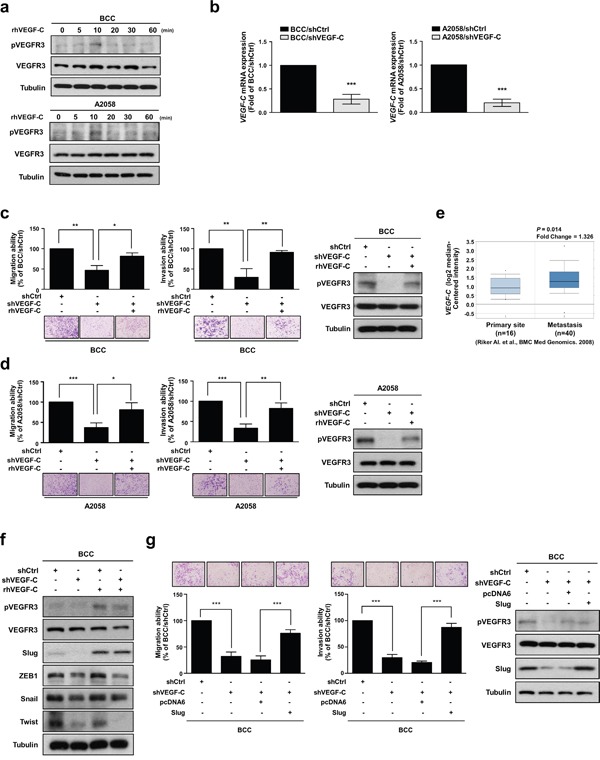
VEGF-C increases the migration and invasion abilities of skin cancer cells **a**. BCC and A2058 cells were incubated with 200 ng/ml of VEGF-C for the indicated time points, and phosphorylated VEGFR3 and VEGFR3 were detected by Western blot analysis. Tubulin was used as a loading control. **b**. The mRNA expression of VEGF-C was analyzed by real-time qRT-PCR. **c** and **d**. A transwell assay was performed to examine cell migration and invasion abilities. Cell motility and phosphorylation of VEGFR3 was inhibited by knockdown of VEGF-C and recovered by treatment with rhVEGF-C by transwell assay and Western blot analysis, respectively. **e**. Oncomine database analysis showed elevated *VEGF-C* expression in metastatic melanoma tissues. **f**. Expression of EMT-inducing transcription factors (Slug, ZEB1, Twist and Snail) was determined by Western blot analysis. **g**. The effects of Slug expression on skin cancer cell motility were determined by transwell assay. Western blot analysis was used to confirm the expression of the indicated proteins. The results are shown as the mean ± SD of three independent experiments, each performed in triplicate. *P < 0.05, **P < 0.001, ***P < 0.0001 (Student's *t* test).

We first determined the activation of VEGFR3 in two skin cancer cell lines, BCC and A2058, by treating the cells with recombinant human VEGF-C protein (rhVEGF-C). We found that rhVEGF-C protein treatment increased VEGFR3 phosphorylation (pVEGFR3) in BCC and A2058 cells after 10 minutes (Figure 1a). To further investigate whether VEGF-C affects skin cancer cell migration and invasion abilities, we established stable VEGF-C-knockdown cells (BCC/shVEGF-C and A2058/shVEGF-C) and control cells (BCC/shCtrl and A2058/shCtrl), and *VEGF-C* mRNA expression was measured by qRT-PCR analysis (Figure 1b). Knockdown of VEGF-C significantly suppressed the migration and invasion abilities of skin cancer cells (Figure 1c and 1d). Moreover, rhVEGF-C treatment restored the migration and invasion abilities of VEGF-C-knockdown cells (Figure 1c and 1d). An analysis of the Oncomine database also showed that *VEGF-C* is more highly expressed in metastatic sites of skin cancer patients than in primary sites (Figure 1e), indicating that VEGF-C is crucial for skin cancer progression.

Accumulating evidence indicates that the EMT is necessary for tumor progression and malignant transformation, which strongly enhances cancer cell motility [27]. The EMT program is orchestrated by EMT-inducing transcription factors (EMT-TFs) primarily belonging to three families, Snail, Twist and ZEB [28, 29]. Therefore, we investigated whether VEGF-C regulates EMT-TFs and found that VEGF-C depletion decreased the protein expression of Slug, ZEB1 and Twist. Notably, only Slug expression was recovered by treatment with rhVEGF-C (Figure 1f). Slug (also known as Snail2), a member of the Snail family, can repress E-cadherin expression and trigger EMT [30]. Numerous studies have indicated that Slug is involved in malignant transformation and metastatic progression in various cancers [31]. In addition, it has been reported that VEGF-A can induce Slug protein expression in pancreatic carcinoma cells and is positively correlated with *Slug* in patients with glioblastoma [32, 33]. As shown in Table 1, *Slug* expression levels were positively correlated with several clinical-pathologic features, such as metastatic event, melanoma and skin squamous cell carcinoma. To further examine the effects of Slug on VEGF-C/VEGFR-mediated cell motility in skin cancer cells, we overexpressed Slug in VEGF-C-knockdown cells and found that Slug re-expression can recover the shVEGF-C-inhibited cell migration and invasion abilities (Figure 1g). Taken together, these results suggest that VEGF-C/VEGFR3 signaling plays an important role in the enhancement of the migration and invasion abilities of skin cancer cells through up-regulation of Slug.

**Table 1:** Clinicopathological features, clinical outcomes and their association with *Slug* expression in skin cancer datasets

Datasets	Clinicopathological features	*Slugexpression	^†^*P*-value
Laurent (n = 63)	Metastatic Event at 1 Year	1.606	0.025
	Metastatic Event at 3 Years	2.420	8.22E-4
Talantov (n = 70)	Benign Melanocytic Skin Nevus	2.803	6.85E-6
	Cutaneous Melanoma	2.075	2.68E-4
Haqq (n = 37)	Melanoma	4.491	0.004
	Non-Neoplastic Nevus	3.302	0.011
Riker (n = 87)	Skin Squamous Cell Carcinoma	1.817	0.001
	Skin Basal Cell Carcinoma	1.448	0.013
Nindl (n = 15)	Skin Squamous Cell Carcinoma	1.728	0.049

*Fold change (Log2 median-centered intensity)

^†^*P*-value

^‡^Pearson's correlation coefficient of Individual dataset were obtained from the Oncomine database.

### VEGF-C enhances the cancer stemness properties in skin cancer

**Figure 2:**
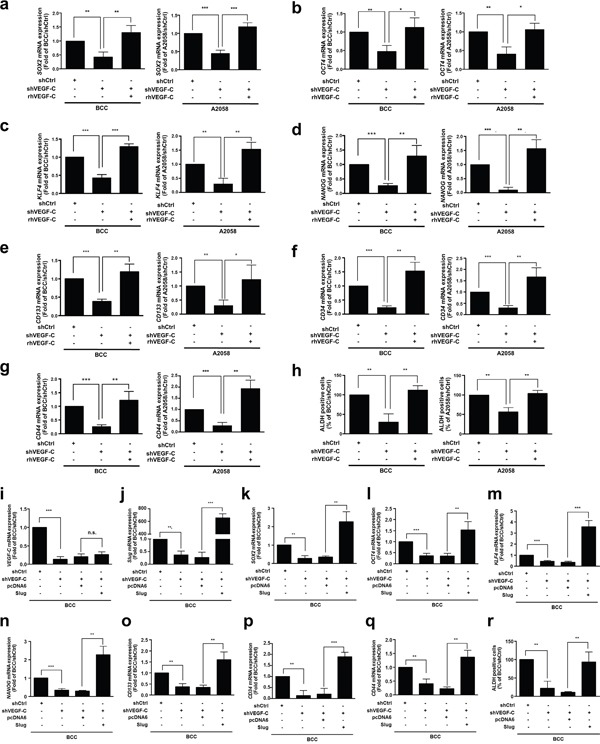
VEGF-C enhances the cancer stemness properties in skin cancer **a-g**. QRT-PCR analysis of the *SOX2*, *OCT4*, *KLF4*, *NANOG*, *CD133*, *CD34* and *CD44* mRNA expression in indicated cells with rhVEGF-C (200 ng/ml) treatment. **h**. ALDH activity of the indicated cells was measured by flow cytometry. **i-q**. Expression of *VEGF-C* (i) and *Slug* (j) was detected, and Slug expression restores the expression of CSC markers (*SOX2* (k), *OCT4* (l), *KLF4* (m), *NANOG* (n), *CD133* (o), *CD34* (p) and *CD44* (q)) in indicated cells by QRT-PCR analysis. **r**. Slug expression recovers ALDH activity in indicated cells by flow cytometry.

Emerging evidence has suggested that EMT inducers not only contribute cell motility but also regulate stem cell properties and cell survival [34–37]. Cancer stem cells (CSCs) have been described as vital mediators of cell mobility in various cancers including lung, breast and skin [26, 38, 39]. Therefore, we examined whether VEGF-C affects cancer stemness in skin cancer cells and found that knockdown of VEGF-C significantly decreased the expression of the CSC markers *SOX2*, *OCT4*, *KLF4*, *NANOG*, *CD133*, *CD34* and *CD44* as well as ALDH activity, which is a hallmark of CSCs (Figure 2a-2h). As expected, rhVEGF-C treatment restored CSC marker expression and ALDH activity in VEGF-C-knockdown cells (Figure 2a-2h). An Oncomine database analysis also demonstrated that *VEGF-C* expression was positively correlated with the expression of some CSC markers, such as *OCT4*, *KLF4*, *NANOG*, *CD44*, *CD34* and *CD133* (Supplementary Figure S1a-S1f), suggesting that VEGF-C increases the CSC features of skin cancer cells.

Next, to determine the effects of Slug on VEGF-C/VEGFR3-mediated cancer stemness in skin cancer cells, we re-expressed Slug in VEGF-C-knockdown BCC cells. The results showed that Slug recovered CSC marker expression and ALDH activity (Figure 2i-2r), suggesting that Slug is a key downstream factor in the VEGF-C/VEGFR pathway.

### VEGF-C increases slug expression, cell migration, invasion and stemness through YAP1

**Figure 3:**
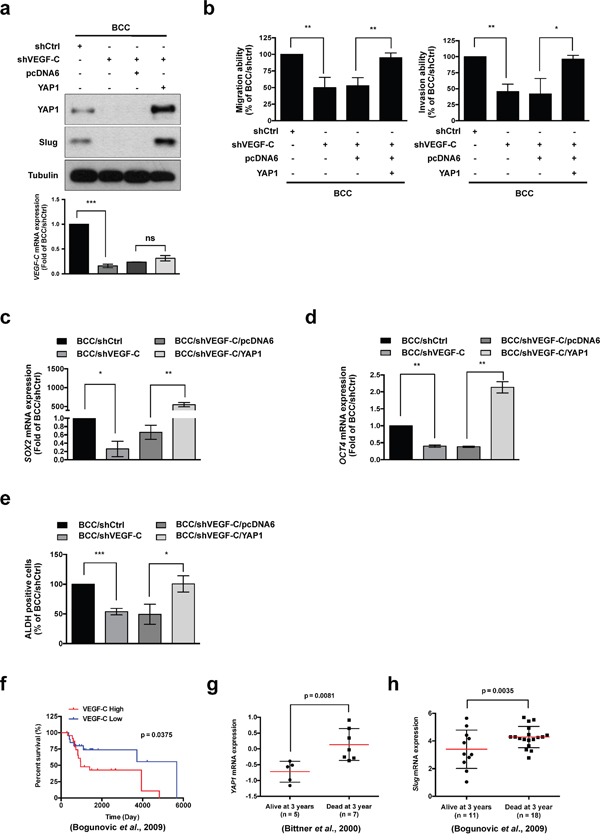
VEGF-C increases Slug expression, cell migration, invasion and stemness through YAP1 **a**. (Upper panel) Western blot analysis of Slug and YAP1 in indicated cells. Tubulin was used as a loading control. (Lower panel) QRT-PCR analysis of *VEGF-C* expression in indicated cells. **b**. A transwell assay demonstrated the migration and invasion abilities of indicated cells. **c** and **d**. QRT-PCR analysis of *SOX2* and *OCT4* mRNA expression in indicated cells. **e**. ALDH activity of indicated cells was determined by flow cytometry. **f-h**. The Oncomine database was used to analyze the correlation of VEGF-C, YAP1 and Slug with survival rate in skin cancer patients. The results are shown as the mean ± SD of three independent experiments, each performed in triplicate. *P < 0.05, **P < 0.001, ***P < 0.0001 (Student's *t* test).

According to our analysis of Oncomine datasets, we found that *YAP1* was highly expressed in metastatic skin cancer tissues (Supplementary Figure S2a). We next verified the relationship between VEGF-C, YAP1 and Slug by overexpression of YAP1 in VEGF-C-knockdown BCC cells and found that YAP1 restored the shVEGF-C-suppressed Slug expression (Figure 3a). Interestingly, depletion of VEGF-C also decreased YAP1 expression, whereas YAP1 did not affect the *VEGF-C* expression (Figure 3a and Supplementary Figure S2b). Online datasets further demonstrated a positive correlation between *YAP1* and *Slug*, also known as *SNAI2* (Supplementary Figure S2c-S2g). Moreover, YAP1 overexpression improved the migration and invasion abilities that had been abrogated by shVEGF-C in BCC cells (Figure 3b).

We further elucidated whether CSC markers and ALDH activity are affected by YAP1 in BCC cells. Loss of VEGF-C expression diminished the expression of the CSC markers *SOX2* and *OCT4*, consistent with a reduction in ALDH activity, whereas YAP1 overexpression abolished these inhibitory effects (Figure 3c-3e). Analysis of the clinical data in the Oncomine datasets also demonstrated a positive correlation between *YAP1* and CSC markers such as *OCT4*, *SOX2*, *NANOG*, *KLF4*, *ALDHA1*, *CD44* and *CD133* (Table 2). Consistently, the expression of *Slug* was positively correlated with the expression of CSC markers such as *CD44*, *NANOG*, *ALDH1A1* and *KLF4* in skin cancer (Table 3). Most importantly, the analysis of online datasets indicated that high expression of *VEGF-C*, *YAP1* and *Slug* was associated with survival rate in skin cancer patients (Figure 3f-3h). Taken together, these results demonstrated that the VEGF-C/VEGFR3 signaling pathway affects cell mobility, cancer stemness properties and progression by the regulation of Slug through YAP1 in skin cancer.

**Table 2:** The association between stem cell markers and *YAP1* expression in skin cancer datasets

Stem cell markers	Datasets	^†^Pearson correlation, *r*	^‡^*P*-value
*OCT4*	Riker (n = 87)	0.3445	0.0011
*SOX2*	Beasley (n = 28)	0.5504	0.0024
	Nindl (n = 15)	0.5191	0.0474
*NANOG*	Riker (n = 87)	0.4529	< 0.0001
*KLF4*	Riker (n = 87)	0.5593	< 0.0001
	Talantov (n = 70)	0.3372	0.0043
*ALDH1A1*	Beasley (n = 28)	0.5799	0.0012
	Bogunovic (n = 44)	0.3364	0.0256
*CD44*	Bittner (n = 31)	0.5683	0.0009
	Bogunovic (n = 44)	0.4901	0.0007
	Riker (n = 87)	0.2940	0.0057
	Smith (n = 18)	0.5956	0.0091
	Talantov (n = 70)	0.4553	< 0.0001
	Xu (n = 83)	0.3048	0.0051
	Nindl (n = 15)	0.7799	0.0006
*CD133*	Smith (n = 18)	0.6350	0.0046

^†^*r*, Pearson's correlation coefficient and ^‡^*P*-value for two-tailed Student's *t* test of Individual dataset (Oncomine database). *P* value < 0.05 was considered statistically significant.

**Table 3:** The association between stem cell markers and *Slug* expression in skin cancer datasets

Stem cell markers	Datasets	^†^Pearson correlation, *r*	^‡^*P*-value
*CD44*	Beasley (n = 28)	0.6761	< 0.0001
	Bogunovic (n = 44)	0.4893	0.0007
	Haqq (n = 28)	0.4985	0.0069
	Harlin (n = 44)	0.3852	0.0098
	Riker (n = 86)	0.2308	0.0325
	Xu (n = 82)	0.3626	0.0008
	Talantov (n = 70)	0.6651	< 0.0001
*NANOG*	Beasley (n = 28)	0.4171	0.0272
	Harlin (n = 44)	0.3198	0.0343
	Riker (n = 86)	0.3112	0.0035
*ALDH1A1*	Bogunovic (n = 44)	0.3042	0.0447
*KLF4*	Riker (n = 86)	0.4082	< 0.0001

^†^*r*, Pearson's correlation coefficient and ^‡^*P*-value for two-tailed Student's *t* test of Individual dataset (Oncomine database). *P* value < 0.05 was considered statistically significant.

### YAP1 is regulated by VEGF-C via the RAS/MAPK pathway

**Figure 4:**
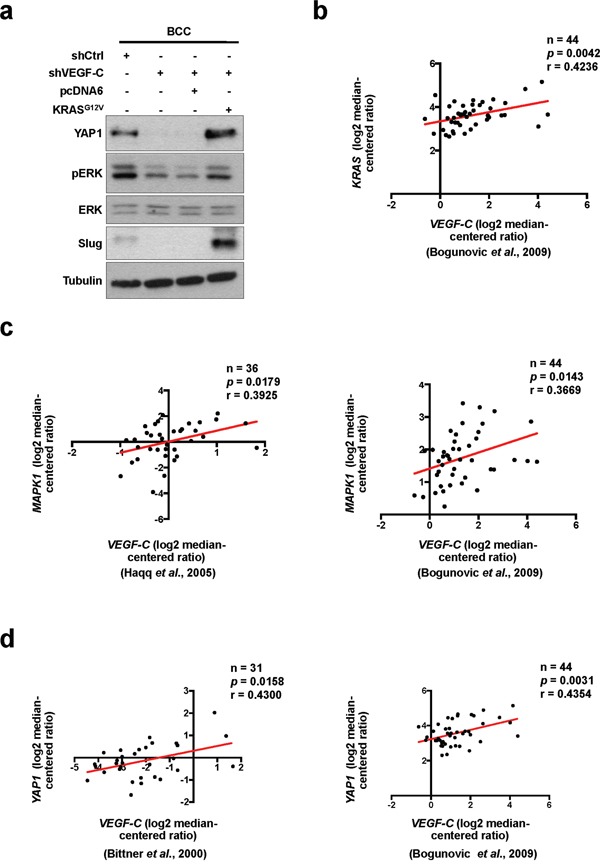
YAP1 is regulated by VEGF-C via the RAS/MAPK pathway **a**. Western blot analysis of indicated proteins in VEGF-C-knockdown cells with or without KRAS^G12V^ overexpression. **b-d**. The Oncomine dataset analysis showed a positive correlation of *VEGF-C* with *KRAS* (b), *MAPK1* (c) and *YAP1* (d) in skin cancer tissues.

YAP1 was previously defined as a key effector of oncogenic RAS/MAPK signaling, which promotes cell proliferation and motility [24, 40]. We next assessed whether RAS is involved in VEGF-C-regulated YAP1 expression. The phosphorylation of ERK was decreased in VEGF-C-knockdown BCC cells, but recovered by overexpression of constitutively activated KRAS^G12V^ [41, 42] (Figure 4a). Moreover, KRAS^G12V^ abrogated the reduction of YAP1 and Slug expression driven by VEGF-C knockdown (Figure 4a). To address the clinical relevance of VEGF-C, YAP1, Slug and the RAS/MAPK pathway in clinical samples, the public database showed that *VEGF-C* was positively correlated with *KRAS*, *MAPK1* and *YAP1* expression (Figure 4b-4d). Furthermore, expression of *Slug* was positively correlated with *KRAS* and *MAPK1* expression in skin cancer (Supplementary Figure S3 and S4). *KRAS* expression was also significantly correlated with clinical-pathologic features such as metastatic event and skin cancer (Table 4). Consequently, these results indicate that VEGF-C regulates YAP1 and Slug expression via the RAS/MAPK pathway to enhance cell mobility and cancer stemness in skin cancer cells.

**Table 4:** Clinicopathological features, clinical outcomes and their association with *KRAS* expression in skin cancer datasets

Datasets	Clinicopathological features	*KRAS expression	^†^*P*-value
Xu (n = 154)	Metastasis	1.527	1.67E-7
Laurent (n = 63)	Metastatic Event at 1 Year	1.511	0.041
Talantov (n = 70)	Benign Melanocytic Skin Nevus	1.650	4.32E-4
	Cutaneous Melanoma	1.619	6.51E-4
Nindl (n = 15)	Skin Squamous Cell Carcinoma	3.039	0.020
Riker (n = 87)	Skin Squamous Cell Carcinoma	1.641	0.002
	Skin Basal Cell Carcinoma	1.380	0.010

*Fold change (Log2 median-centered intensity)

^†^*P*-value

^‡^Pearson's correlation coefficient of Individual dataset were obtained from the Oncomine database.

### Anti-VEGFR3 peptide represses VEGF-C-induced signaling, cell mobility and cancer stemness in skin cancer

**Figure 5:**
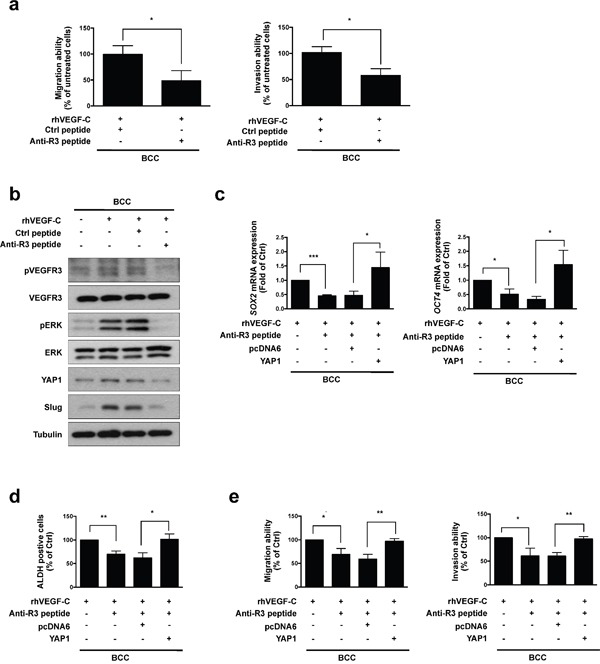
Anti-VEGFR3 peptide represses VEGF-C-induced signaling, cell mobility and cancer stemness in skin cancer **a**. A transwell assaydemonstrated the migration and invasion abilities in BCC cells pre-incubated with rhVEGF-C followed by anti-VEGFR3 (anti-R3) or control (Ctrl) peptide treatment. **b**. Western blot analysis of indicated proteins in rhVEGF-C-pre-incubated cells treated with anti-R3 or Ctrl peptide. **c**. qRT-PCR analysisof *SOX2* and *OCT4* mRNA expression in indicated cells. **d**. ALDH activity in indicated cells was analyzed by flow cytometry. **e**. A transwell assay showed migration and invasion abilities in indicated cells. The results are shown as the mean ± SD of three independent experiments, each performed in triplicate. *P < 0.05, **P < 0.001, ***P < 0.0001 (Student's *t* test).

In our previous study, we demonstrated that a small peptide specifically inhibits VEGF-C/VEGFR3-mediated signaling, migration and invasion in cancer cells [26]. We observed that the anti-VEGFR3 peptide significantly blocked the rhVEGF-C-induced migration and invasion abilities of BCC cells (Figure 5a). We further analyzed the effects of the anti-VEGFR3 peptide on VEGF-C induced signaling and found that treatment with the anti-VEGFR3 peptide abolished the rhVEGF-C-induced phosphorylation of VEGFR3 and ERK. VEGF-C-induced YAP1 and Slug expression were also repressed by the anti-VEGFR3 peptide (Figure 5b). Since YAP1 acts as a critical downstream mediator of VEGF-C/VEGFR3 signaling, we overexpressed YAP1 in BCC cells and found that YAP1 restored anti-VEGFR3 peptide-inhibited *SOX2* and *OCT4* expression and ALDH activity, as well as migration and invasion abilities (Figure 5c-5e). These data confirm that the anti-VEGFR3 peptide abolishes activation of the VEGF-C/VEGFR3-KRAS/MAPK signaling pathway to decrease the YAP1-mediated Slug expression, cancer stemness, migration and invasion of skin cancer cells.

## DISCUSSION

**Figure 6:**
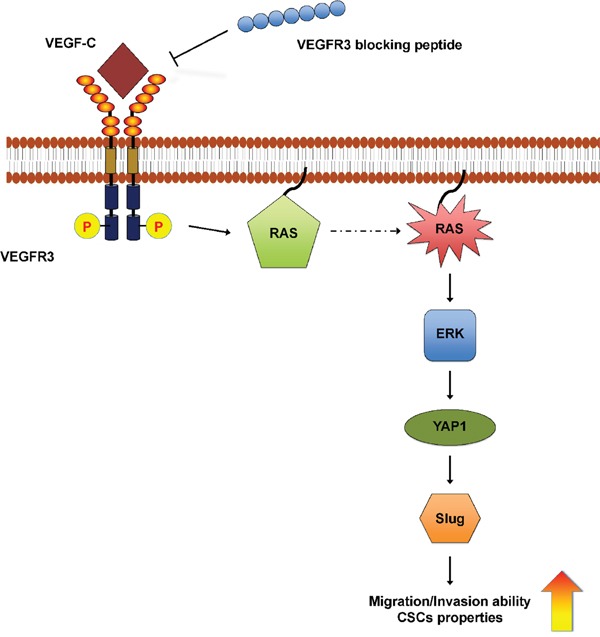
A schematic model of the VEGF-C/VEGFR3-mediated KRAS/MAPK-YAP1/Slug pathway in skin cancer progression In skin cancer cells, VEGF-C/VEGFR3 signaling activates the KRAS/MAPK axis to up-regulate YAP1 and Slug expression, further enhancing cell mobility and cancer stemness. Anti-VEGFR3 peptide functions as a repressor and could be a therapeutic strategy.

Elevated YAP1 expression has been shown to induce cell proliferation and ECM remodeling through CCN1 and CCN2 expression in BCC [43]. Consistent with previous findings, we further demonstrated that VEGF-C-mediated YAP1 expression increases Slug expression and contributes to the migration and invasion abilities of skin cancer cells. Previous reports have indicated that *YAP1* expression not only positively correlates with the expression of *OCT4* and *SOX2* and is involved in self-renewal properties and differentiation of embryonic stem cells [44] but also promotes oncogenic transformation by interfering with the Hippo signaling pathway [45]. It has recently been proposed that migrating cancer cells that contain stem-like properties might divide asymmetrically; one daughter cell starts proliferation and differentiation, the remaining cell can either undergoes a new asymmetric division or produces metastasis [46]. In the present study, we showed that VEGF-C-induced YAP1 expression modulates Slug expression to enhance cell migration and invasion, which further increases cancer stemness to promote skin cancer progression (Figure 6), suggesting that regulation of YAP1 is crucial for studying tumorigenicity of skin cancer and is a potential target of therapeutic strategies.

We have successfully developed an anti-VEGFR3 peptide that blocks VEGF-C/VEGFR3 axis-induced tumor initiation, migration, invasion and chemoresistance [26], suggesting that the anti-VEGFR3 peptide has the potential to serve as a novel therapy for skin cancer. In a previous study, high VEGF-C expression in patients with metastatic melanoma was shown to correlate with lymph node localization [18]. Elevated VEGF-C leads to enhanced tumor lymphangiogenesis and metastasis in SCC [47], suggesting that VEGF-C may be a biomarker of tumor metastasis and serve as a novel therapeutic target in the treatment of skin cancer. A recent study indicates that VEGF-C/VEGF-D regulate the early stages of skin cancer progression and inhibition of VEGF-C/VEGF-D by a soluble Fc-fused form of VEGFR3 significantly suppressed chemical-induced SCC carcinogenesis in an animal model *in vivo* [48]. Consistently, our findings demonstrate that the novel and simply prepared anti-VEGFR3 peptide is able to significantly block the VEGF-C/VEGFR3 pathway, which further inhibits cancer stemness, migration and invasion in skin cancer cells.

BCC is the most common non-melanoma skin cancer in humans, which morbidity is growing annually worldwide. In some cases, BCC can be highly invasive due to genetic alterations in the Hedgehog (HH) signaling pathway [3]. Although several pathways associated with BCC are known, the mechanisms involved in the morphology, aggressiveness and response to treatment in BCC still need to be elucidated. KRAS activation regulates many cell properties, such as cell growth, division and survival, through many critical signaling pathways [49]. Significant evidence suggests that mutation of KRAS causes constitutive activation and persistent stimulation of downstream signaling pathways, and this phenomenon can be found in many types of cancer [50–52]. An increasing number of studies also indicate that KRAS mutations (G12C or G12D) are required for BCC, SCC and melanoma development [53–55]. Bonilla *et al*. showed that mutations in the RTK-RAS-PI3K pathway are involved in BCC tumor progression [56]. Moreover, KRAS is a critical regulator for the maintenance of mesenchymal phenotypes and metastatic ability in skin, breast, colon and pancreatic cancers [40, 57–59]. KRAS-mediated Slug expression is necessary for the EMT processes [40, 58]. Interestingly, KRAS has been reported to regulate the activity of YAP1 through the MAPK pathway [24]; however, a link between VEGF-C, KRAS and YAP1 has not been established. Our study confirms that VEGF-C is a critical inducer of cancer stemness, metastasis and invasion in skin cancer through regulating YAP1 expression via KRAS/MAPK signaling.

VEGF-A is reported to be an inducer of EMT through enhancing Snail expression in breast cancer cells, partly by inhibiting GSK3β activity [60, 61]. VEGF-C can induce vimentin and N-cadherin expression and suppress cytokeratins and E-cadherin expression, which are common markers used for the detection of EMT [62]. In the present study, we demonstrate that VEGF-C induces Slug expression to increase EMT in skin cancer. EMT is also suggested to confer CSC qualities. In the tumor microenvironment, several soluble factors are indicated to orchestrate EMT and induce CSC production from differentiated cancer cells [63]. For example, HGF in colon cancer cells [64] and TGF-β in mammary epithelial cells [65] can induce the EMT-CSC program. Our findings exhibit that VEGF-C can induce CSC features in skin cancer cells (Figure 2). However, it is not clear whether VEGF-C induces EMT and cancer stemness through identical or similar molecular pathway, or if the regulation of EMT-TFs modulates CSC features.

In summary, our study reveals a novel mechanism by which the VEGF-C/VEGFR3 axis regulates Slug expression in skin cancer through the KRAS/MAPK-YAP1 pathway and contributes to the cancer stemness and metastasis of skin cancer. Moreover, these findings suggest that targeting the VEGFR3 signaling pathway with an anti-VEGFR3 peptide may be a potential therapeutic strategy for skin cancer treatment.

## MATERIALS AND METHODS

### Antibodies and reagents

Recombinant human VEGF-C protein (rhVEGF-C) was purchased from Sino Biological (Beijing, China). Western blot analyses were performed with the following antibodies: VEGFR3 (LifeSpan BioSciences, WA, USA), p-VEGFR3 (Y1063/1068, Cell Applications, CA, USA), YAP1 (#4912, Cell signaling, Beverly, MA, USA), p44/p42 MAP Kinase (ERK) (#9102, Cell signaling, Beverly, MA, USA), Phospho-p44/p42 MAPK (p-ERK) (Thr202/Tyr204, #4377, Cell signaling, Beverly, MA, USA), Vimentin (Neomarkers, Fremont, CA, USA), Snail (GTX81086, GeneTex, San Antonio, TX, USA), Slug (#9585, Cell signaling, Beverly, MA, USA), Twist (sc-81417, Santa Cruz Biotechnology, Inc.) and α-Tubulin (T5168, Sigma-Aldrich, St. Louis, MO, USA). The sequences of the peptides selected by binding to VEGFR3 are as follows: Ctrl peptide (peptide 4: CNDSHMKLC) and anti-VEGFR3 (anti-R3) peptide (peptide 5: CNYSHLFYC) [26].

### Cell culture and transfection

The human basal cell carcinoma cell line BCC was a kind gift from Dr. Min-Liang Kuo [66], the human melanoma cell line A2058 (ATCC CRL-11147) was kindly provided by Dr. Wun-Shaing Chang [67], and the human embryonic kidney cell line 293T (ATCC CRL-3216) was purchased from the American Type Culture Collection (ATCC, Manassas, VA, USA). BCC cells were cultured in RPMI/1640 medium, A2058 cells were cultured in DMEM/F12 (1:1) medium, and 293T cells were cultured in high-glucose/DMEM medium. All cells were incubated at 37°C in a humidified 5% CO_2_ atmosphere, and the media were supplemented with 10% fetal bovine serum (FBS, (Carlsbad, CA, USA) and 1% penicillin/streptomycin (Carlsbad, CA, USA).

### Plasmid and shRNA clones

The YAP1 expression plasmid was purchased from Bioresource Collection and Research Center, Hsinchu, Taiwan. For establishment of the VEGF-C-knockdown skin cancer cells, lentiviral VEGF-C shRNA clones TRCN0000058505 and TRCN0000058507 were purchased from National RNAi Core Facility at Academia Sinica, Taipei, Taiwan.

### Lentivirus production and infection

Recombinant lentiviruses were packaged according to a previously described method [39]. Briefly, HEK293T cells were co-transfected with mixtures of the indicated expressing plasmid, the pCMV plasmid and the packaging plasmid (pVSVG) using PEI (Sigma-Aldrich). The viruses were collected from the culture medium on day 2 after transfection and filtered with a 0.45-μm filter. For lentivirus infection, the BCC and A2058 cells were infected with 3 ml lentivirus supernatant and 2 μl of polybrene (Hexadimethrine bromide, Sigma) and after 48 hours the stably transduced cells were selected by treatment with 5 μg/ml puromycin.

### RNA Isolation and reverse transcription and quantitative RT-PCR

Total RNA was isolated using Trizol (Invitrogen) and cDNAs were reverse transcribed by M-MLV reverse transcriptase (Invitrogen) according to the manufacturer's instructions. Quantitative real-time PCR was performed for at least three independent experiments, each performed in triplicate, by the LightCycler 480 (Roche). For mRNA detection, PCR reactions contained 2 μl of cDNA, 0.5 μM of each forward and reverse primer, 1 μM Universal ProbeLibrary Probe (Roche) and 1× LightCycler TaqMan Master mix. Amplification curves were generated with an initial denaturing step at 95°C for 10 minutes, followed by 50 cycles of 95°C for 5 seconds, 60°C for 10 seconds, and 72°C for 1 second. The *GAPDH* gene was used as an internal control. The PCR primers for the gene expression analysis are as follows: *VEGF-C* (forward primer: tgccagcaacactaccacag and reverse primer: gtgattattccacatgtaattggtg), *SOX2* (forward primer: atgggttcggtggtcaagt and reverse primer: actctggggctccttcttg), *OCT4* (forward primer: gaggcaacctggagaatttg and reverse primer: cagaaccacactcggacca), and *GAPDH* (forward primer: agccacatcgctcagacac and reverse primer: gcccaatacgaccaaatcc). The relative levels of gene expression were calculated by 2^−ΔCT^ = CT of the target gene – CT of the *GAPDH* gene.

### Western blotting analysis

Western blot analysis was performed as previously described [68]. Briefly, cells were washed twice with PBS and lysed with RIPA lysis buffer (20 mM Tris-HCl, pH 8.0, 150 mM NaCl, 0.5% Nonidet P-40, 1 mM EDTA, 10 mM Na_3_VO_4_ and 1× protease inhibitor cocktail (Sigma)). Cell lysates were sonicated for 1 minute and then centrifuged at 13,000 ×*g* for 30 minutes. An equal amount of total protein was separated by SDS-PAGE and transferred to PVDF membranes. Primary antibodies were incubated overnight at 4°C and followed by the appropriate secondary antibodies conjugated to horseradish peroxidase. The indicated proteins were visualized using ECL reagents (Millipore Forte (WBLUF0500); Millipore Crescendo (WBLUR0500)).

### Transwell migration and invasion assays

Transwell assays were performed as described previously [26]. A2058 cells (2 × 10^4^ cells) or BCC cells (3 × 10^4^ Cells) were seeded in the upper well (Corning Costar; Lowell, MA, USA) with serum-free medium for the migration assay or a matrigel-coated membrane for the invasion assay, followed by addition of 10% FBS-containing medium in the lower well. The images of cells migrating or invading through the lower membrane were taken by light microscope.

### ALDH activity assay

ALDH enzymatic activity was determined by the Aldefluor kit (StemCell Technologies, Vancouver, BC, Canada) according to the manufacturer's instructions. ALDH-positive cells were detected in the green fluorescence channel (FL1) by flow cytometry analysis. The specific ALDH inhibitor (diethylaminobenzaldehyde (DEAB))-treated cells were used as the control to set the gates defining the ALDH-positive region. The results are representative of three independent experiments, each performed in triplicate.

### Cell proliferation assay

Cell proliferation was examined by MTT assay. Cells (5 × 10^3^) were seeded in 96-well culture plates for the indicated time points, and then incubated with 5 mg/ml MTT reagent. After 4 hours, the supernatants were removed and 100 μl DMSO was added to dissolve the crystals. The absorbance in each well was measured at a wavelength of 570 nm with background subtraction at 630 nm by an ELISA reader.

### Statistical analysis

All statistical analyses were performed with Prism 6 software (La Jolla, CA). Data were analyzed as the mean ± SD. The two-tailed Student's *t* test was used to analyze the means of the different two groups. A *P* value of less than 0.05 was considered statistically significant.

## SUPPLEMENTARY FIGURES



## Abbreviations

VEGF-C: Vascular endothelial growth factor-C
EMT: epithelial-mesenchymal transition
MAPK: mitogen-activated protein kinases
YAP1: yes-associated protein 1
VEGFR3: VEGF receptor 3
SCC: squamous cell carcinoma
BCC: basal cell carcinoma
MBCC: metastatic BCC
PlGF: placental growth factor
rhVEGF-C: recombinant human VEGF-C
EMT-TFs: EMT-inducing transcription factors
CSCs: cancer stem cells
HH: Hedgehog.
