# Supplementary material for: Targeting the VEGF-C/VEGFR3 axis suppresses Slug-mediated cancer metastasis and stemness via inhibition of KRAS/YAP1 signaling
Source: Oncotarget. 2016 Nov 25;8(3):5603–18. doi: 10.18632/oncotarget.13629 (PMC5354933; doi:10.18632/oncotarget.13629)
Supplement: Supplementary file 1 [file oncotarget-08-5603-s001.pdf]

Targeting the VEGF-C/VEGFR3 axis suppresses Slug-mediated cancer metastasis and stemness via inhibition of KRAS/YAP1 signaling

SUPPLEMENTARY FIGURES

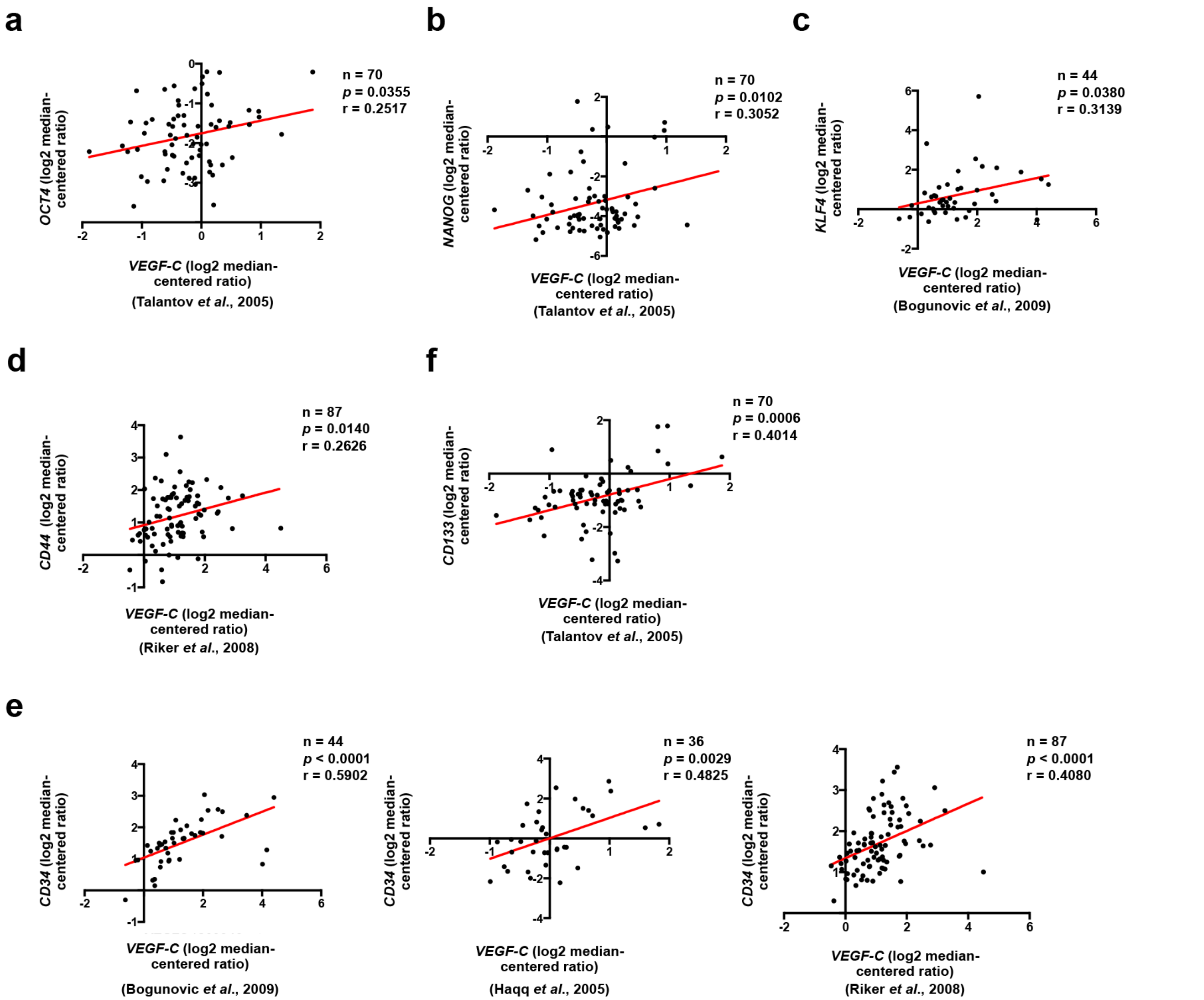

Supplementary Figure S1: a-f. The Oncomine database analysis indicated that the *VEGF-C* expression in skin cancer was positively correlated with the expression of cancer stem cell markers such as *OCT4* (a), *NANOG* (b), *KLF4* (c), *CD44* (d), *CD34* (e) and *CD133* (f).

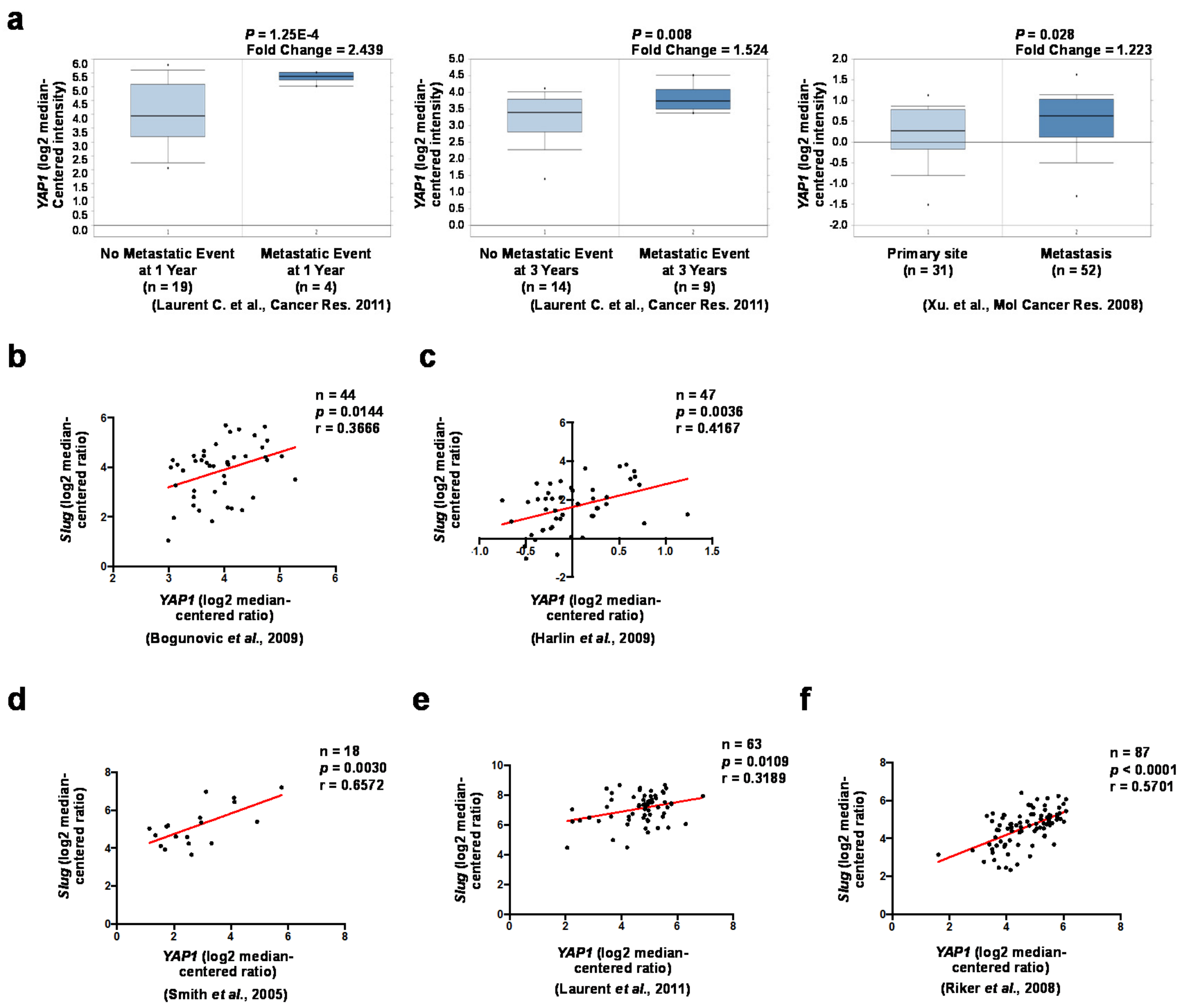

Supplementary Figure S2: a. The Oncomine database analysis indicated that *YAP1* is highly expressed in metastatic skin cancer tissues. **b-f.** The Oncomine database analysis indicated that *YAP1* expression positively correlates with *Slug* expression in skin cancer tissues.

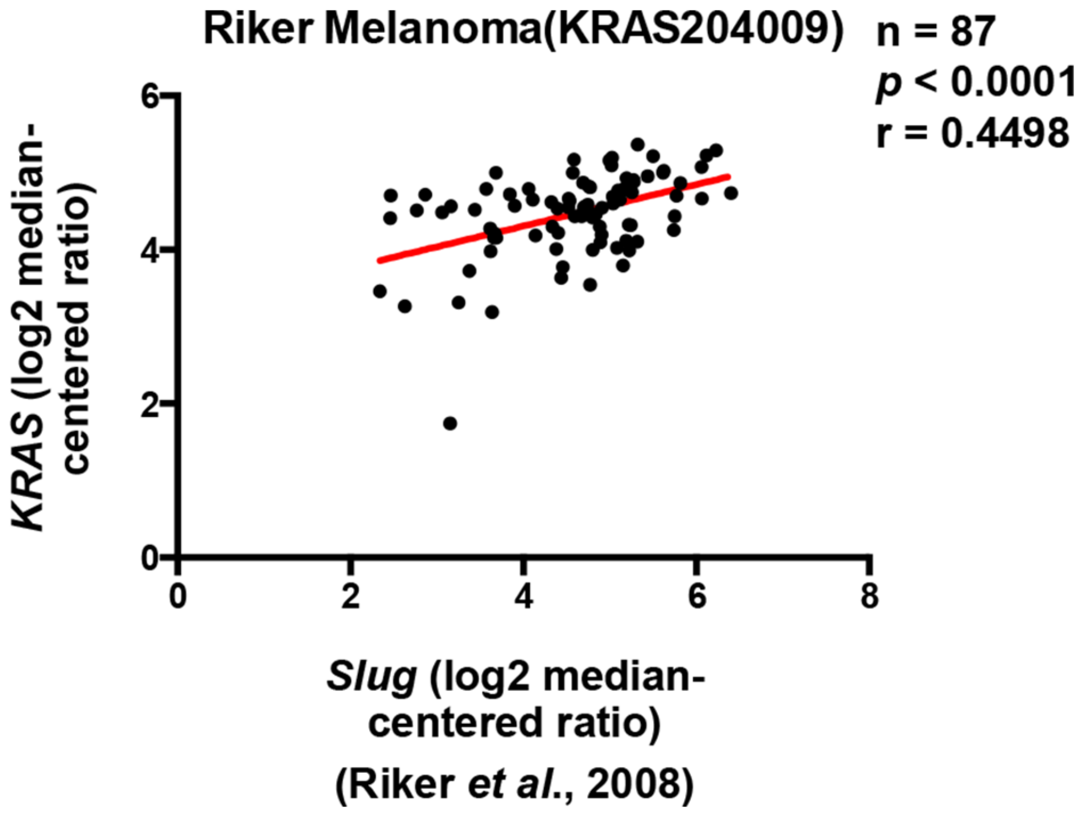

Supplementary Figure S3: The Oncomine database analysis indicated that *KRAS* expression positively correlates with *Slug* expression in skin cancer tissues.

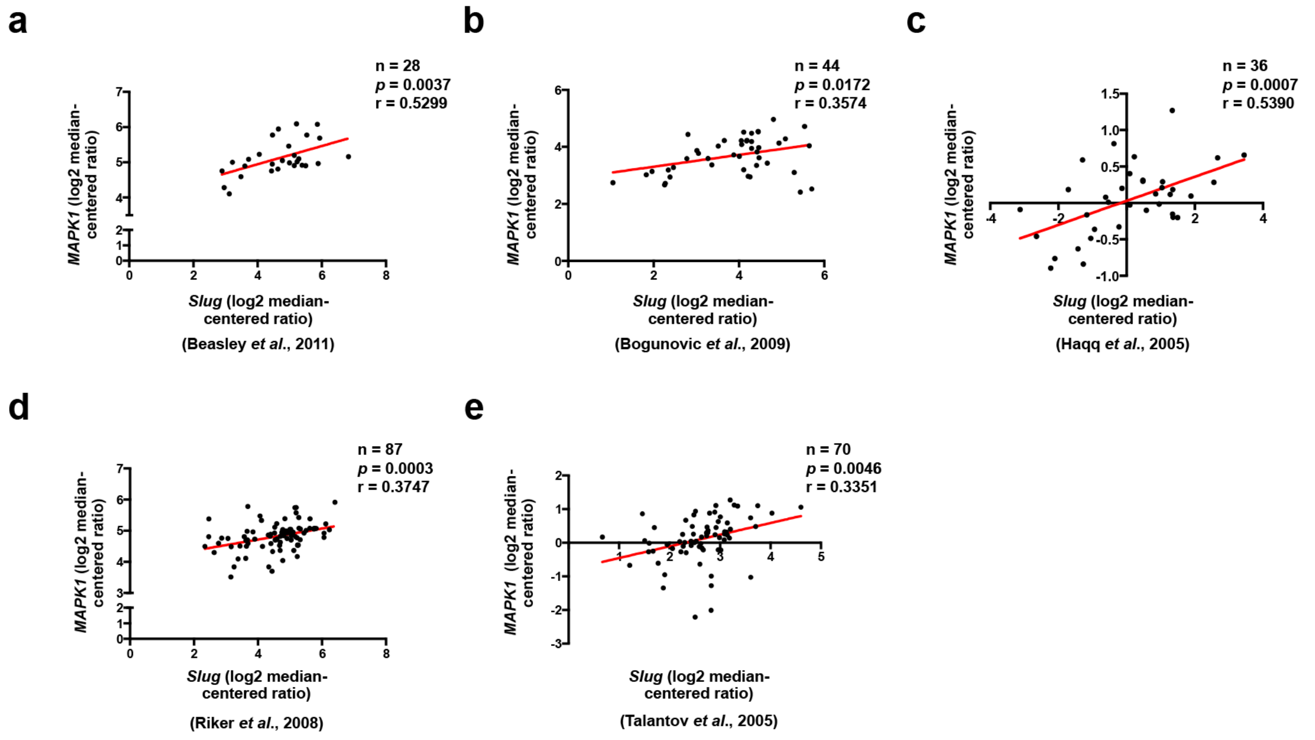

Supplementary Figure S4: The Oncomine database analysis indicated that *MAPK1* expression positively correlates with *Slug* expression in skin cancer tissues.
